# Supplementary material for: Arabic translation and cultural adaptation of a training load and player monitoring in high-level football questionnaire: A cognitive interview study
Source: PLoS One. 2024 Apr 17;19(4):e0302006. doi: 10.1371/journal.pone.0302006 (PMC11023223; doi:10.1371/journal.pone.0302006)
Supplement: S1 Questionnaire — (DOCX) [file pone.0302006.s001.docx]

**الأسئلة**

| عدد سنوات الخبرة في العمل مع فرق كرة القدم ذات المستويات العليا: |
| --- |
| المسمى الوظيفي: |
| النادي: |

**في أي دوري ينافس فريقك حالياً؟**

| دوري روشن السعودي |  |
| --- | --- |
| دوري يلو للدرجة الأولى |  |
| أخرى (يرجى التحديد): | |

**الهيكل التنظيمي للفريق**

يرجي تحديد عدد الأشخاص العاملين في قسم الطب الرياضي بناديك، بالإضافة لنوع دوامهم (دوام جزئي - دوام كامل - استشاري). إذا كان أحد الأخصائيين العاملين في الفريق يعمل في مهمتين يمكن الإشارة إلى ذلك في العمود الأخير، مثال أخصائي علوم رياضة ومدرب لياقة بدنية.

|  | عدد الموظفين | دوام كامل / دوام جزئي / استشارات؟ |
| --- | --- | --- |
| طبيب رياضي |  |  |
| أخصائي علاج طبيعي |  |  |
| أخصائي فسيلوجيا الجهد البدني/ علوم رياضة |  |  |
| مدرب لياقة بدنية (ميداني) |  |  |
| مدرب قوة عضلية وإعداد البدني |  |  |
| أخصائي تغذية |  |  |
| أخصائي ميكانيكا حيوية |  |  |
| أخصائي علم النفس |  |  |
| أخصائي تحليل بيانات |  |  |

**الأهداف**

يرجى ذكر الأهداف الرئيسية لمراقبة الأحمال التدريبية بناديك، بحيث تذكر الأهداف الأهم أولا ثم الأقل أهمية. يمكنك ذكر ما يصل إلى خمس أهداف:

| 1 |
| --- |
| 2 |
| 3 |
| 4 |
| 5 |

**مراقبة التدريبات**

يرجى اختيار جميع الأدوات والأجهزة التي تستعملها لمراقبة **أحمال التدريبات** من القائمة التالية. يرجى اختيار جميع ما يتم استخدامه. *يمكن إضافة معلومات إضافية في مربع النص أدناه*

| أدوات التحليل الزمني للحركه (مثال GPS، الرجاء ذكر أي الأنظمة أدناه) |  |
| --- | --- |
| أجهزة مراقبة معدل ضربات القلب |  |
| أدوات قياس التسارع (تشمل تلك المدمجة مع وحدات GPS) |  |
| مقياس بورغ للإحساس بالجهد (RPE) |  |
| مقياس تقديري آخر (يرجى ذكره أدناه) |  |
| استخدم هذا المربع لكتابة المعلومات الإضافية | |

يرجى اختيار العبارة التي تصف طريقتكم في **مراقبة أحمال التدريبات في النادي** من القائمة التالية. الرجاء استخدام مربع النص أدناه لإضافة مزيد من التفاصيل إذا لزم الأمر.

| نقوم بجمع بيانات الأحمال التدريبية لكل لاعب على حدة في كل حصة تدريبية. |  |
| --- | --- |
| نقوم بجمع بيانات الأحمال التدريبية لمجموعة محددة من اللاعبين في كل حصة تدريبية (يرجى ذكر السبب أدناه، على سبيل المثال، لا يوجد وحدات GPS كافية لكل لاعب). |  |
| نقوم بجمع بيانات الأحمال التدريبية لكل لاعب على حدة، ولكن ليس في جميع الحصص التدريبية. |  |
| نقوم بجمع بيانات الأحمال التدريبية لمجموعة محددة من اللاعبين، ولكن ليس في جميع الحصص التدريبية. |  |
| استخدم هذا المربع لكتابة المعلومات الإضافية | |

**مراقبة المباريات**

يرجى اختيار جميع الأدوات والأجهزة التي تستعملها لمراقبة **أحمال** **المباريات** من القائمة التالية. يرجى اختيار جميع ما يتم استخدامه. *يمكن إضافة معلومات إضافية في مربع النص أدناه*

| أدوات التحليل الزمني للحركه (مثال GPS، الرجاء ذكر أي الأنظمة أدناه) |  |
| --- | --- |
| اجهزة مراقبة معدل ضربات القلب |  |
| أدوات قياس التسارع (تشمل تلك المدمجة مع وحدات GPS) |  |
| مقياس بورغ للإحساس بالجهد (RPE) |  |
| مقياس تقديري آخر (يرجى ذكره أدناه) |  |
| استخدم هذا المربع لكتابة المعلومات الإضافية | |

**أدوات تقييم الحالة التدريبية**

يرجى اختيار جميع الطرق أو الأدوات (إن وجدت) التي يتم استخدامها في النادي لقياس استجابة اللاعبين للتدريب وتحديد عدد مرات استخدامها.

|  | **يومياً** | **>3 مرات**  **في الاسبوع** | **<3 مرات في الاسبوع** | **مرة واحدة**  **في الاسبوع** | **شهرياً** | **ابداً** | **أخرى (يرجي التحديد)** |
| --- | --- | --- | --- | --- | --- | --- | --- |
| اختبار أداء الأقصى |  |  |  |  |  |  |  |
| اختبار أداء دون الأقصى |  |  |  |  |  |  |  |
| استبانة تقييم ذاتي |  |  |  |  |  |  |  |
| تحاليل دم |  |  |  |  |  |  |  |
| تحليل اللعاب |  |  |  |  |  |  |  |
| تغيرات معدل ضربات القلب |  |  |  |  |  |  |  |
| تدريبات أداء معياريه |  |  |  |  |  |  |  |
| أداء المباراة |  |  |  |  |  |  |  |
| أخرى |  |  |  |  |  |  |  |

**أدوات تقييم الحالة التدريبة**

إذا كنت تقوم بتقييم الحالة التدريبية للاعبين بشكل دوري عن طريق استخدام مقاييس فسيولوجية / أداء / لياقة بدنية / أداء مباريات، نأمل منك تزويدنا ببعض التفاصيل التالية:

1- المقياس المستخدم (على سبيل المثال: 5 دقائق دون الأقصى، عدو مكوكي مسافة 20م بسرعة 12كم/س)

2- تكرار الاستخدام. (على سبيل المثال: مرة في الاسبوع، اليوم الثاني بعد المباراة)

3- المتغيرات التي يتم اختبارها (على سبيل المثال: استرداد معدل ضربات القلب)

4- تحديد ما إذا كان هذا المقياس قد تم تطويره ذاتياً أو تم أخذه من الدراسات العلمية.

نعلم أن بعض البيانات المطلوبة حساسة جداً، ولكن نضمن لكم أن هذه البيانات سيتم التعامل معها بخصوصية تامة ولغرض البحث العلمي فقط. الغرض الأساسي من جمع هذه المعلومات هو مناقشة وفحص علاقة الاستجابة للجرعة التدريبية في مجتمع الدراسة.

| **1.**  **2.**  **3.**  **4.** |
| --- |

إدارة البيانات

يرجى تحديد الطريقة التي تستخدمها لإدارة وتخزين بيانات الأحمال التدريبية من القائمة التالية. يرجى اختيار جميع ما يتم استخدامه.

| برنامج تجاري متاح |  | ☐ |
| --- | --- | --- |
| برنامج مصمم خصيصاً للفريق |  | ☐ |
| مايكروسوفت اكسل |  | ☐ |
| سجلات ورقية |  | ☐ |
| لا أقوم بحفظ البيانات |  | ☐ |
| أخرى (يرجى التحديد): | | |

**مؤشرات** **بيانات** **التدريبات**

يرجى ذكر المؤشرات التي تستخدمها عند مراقبة وتحليل بيانات **أحمال** **التدريبات**. تستطيع ذكر حتى 10 مؤشرات، على أن يتم ذكر المؤشرات الأكثر أهمية أولاً. الرجاء تحديد العتبة الفارقة المستخدمة في المؤشرات ما أمكن.

على سبيل المثال:

المسافة المقطوعة خلال العدو سريعاً (أكثر من 19كلم/س).

عدد مرات التسارع (أكثر من 2م/ث/ث).

معدل ضربات القلب (أكثر من 90٪ من ضربات القلب القصوى) - بالدقائق

مقاييس بورغ لإحساس بالجهد (RPE)

| 1 |
| --- |
| 2 |
| 3 |
| 4 |
| 5 |
| 6 |
| 7 |
| 8 |
| 9 |
| 10 |

**مؤشرات** **بيانات** **المباريات**

يرجى ذكر المؤشرات التي تستخدمها عند مراقبة وتحليل بيانات **أحمال** **المباريات**. تستطيع ذكر حتى 10 مؤشرات، على أن يتم ذكر المؤشرات الأكثر أهمية أولاً. الرجاء تحديد العتبة الفارقة المستخدمة في المؤشرات ما أمكن.

على سبيل المثال:

المسافة المقطوعة خلال العدو سريعاً (أكثر من 19كلم/س).

عدد مرات التسارع (أكثر من 2م/ث/ث).

معدل ضربات القلب (أكثر من 90٪ من ضربات القلب القصوى) - بالدقائق

مقاييس بورغ لإحساس بالجهد (RPE)

| 1 |
| --- |
| 2 |
| 3 |
| 4 |
| 5 |
| 6 |
| 7 |
| 8 |
| 9 |
| 10 |

**تفسير النتائج**

هل تستخدم تحليلات أو عتبات فارقة محددة لكي تساعد في تفسير نتائج مراقبة الأحمال التدريبية؟ الرجاء ذكر بعض التفاصيل لكيفية تفسير النتائج ما أمكن:

- المتغيرات ذات الأهمية.

- التحليل الاحصائي المستخدم.

- اذا كانت البيانات تحلل بشكل مطلق أو بشكل نسبي.

- اذا كانت المؤشرات يتم تفسيرها نسبةً لباقي أعضاء الفريق، أو لمركز اللعب، أو بشكل فردي.

- إذا كانت هناك فترة زمنية محددة يتم استخدامها (على سبيل المثال: الأحمال التدريبية المتراكمة لمدة 7 أيام أو 14 يوم).

|  |
| --- |

**التواصل**

يرجى تحديد الشخص الذي تقوم بالتواصل معه لمناقشة نتائج مراقبة الأحمال التدريبية ونتائج بيانات الحالة التدريبية للاعبين من القائمة التالية، مع تحديد عدد مرات التواصل.

|  | **يومياً** | **اسبوعياً** | **عند**  **الطلب** | **عند وجود**  **فرصة مناسبة** | **ملاحظات (على سبيل المثال: تقرير ورقي/ بريد إلكتروني)** |
| --- | --- | --- | --- | --- | --- |
| المدير الفني / المدرب الرئيسي |  |  |  |  |  |
| المدربين المساعدين |  |  |  |  |  |
| طبيب النادي |  |  |  |  |  |
| أخصائي العلاج الطبيعي |  |  |  |  |  |
| أحد اللاعبين |  |  |  |  |  |
| شخص آخر |  |  |  |  |  |

**ضبط الأحمال التدريبية**

عدد بالترتيب بعض العوامل التي تؤثر في وصفة الأحمال التدريبية و التوصيات الخاصة بها. (للتوضيح: متى توصي بأن يقوم الفريق أو بعض عناصره بالزيادة أو النقصان في الأحمال التدريبية؟ ولماذا؟)

(1 = الأكثر أهمية)

| 1 |
| --- |
| 2 |
| 3 |
| 4 |
| 5 |

**التأثير المتوقع**

**من وجهة نظرك**، ما مدى فعالية أوعدم فعالية مراقبة الأحمال التدريبية لتحقيق الأهداف التالية:

الرجاء تحديد الإجابة من 1 إلى 10 علماً بأن '1' = غير فعالة تماماً،  و '10' = فعالة جداً.

|  | **1**  **غير فعالة تماماً** | **2** | **3** | **4** | **5** | **6** | **7** | **8** | **9** | **10**  **فعالة جداً** |
| --- | --- | --- | --- | --- | --- | --- | --- | --- | --- | --- |
| تقليل معدل الاصابات |  |  |  |  |  |  |  |  |  |  |
| تحسين الأداء الفردي |  |  |  |  |  |  |  |  |  |  |
| تحسين أداء الفريق |  |  |  |  |  |  |  |  |  |  |

**التأثير الفعلي**

**من خلال عملك في الفريق**، ما مدى فعالية أوعدم فعالية مراقبة الأحمال التدريبية لفريقك لتحقيق الاهداف التالية:

الرجاء تحديد الإجابة من 1 إلى 10 علما بأن '1' = غير فعالة تماماً،  و '10' = فعالة جداً.

|  | **1**  **غير فعالة تماماً** | **2** | **3** | **4** | **5** | **6** | **7** | **8** | **9** | **10**  **فعالة جداً** |
| --- | --- | --- | --- | --- | --- | --- | --- | --- | --- | --- |
| تقليل معدل الاصابات |  |  |  |  |  |  |  |  |  |  |
| تحسين الأداء الفردي |  |  |  |  |  |  |  |  |  |  |
| تحسين أداء الفريق |  |  |  |  |  |  |  |  |  |  |

**معوقات فعالية مراقبة الأحمال**

**فيما يتعلق بعملك في الفريق حالياً**، ما هي العوامل التي تعتبرها أكبر العوامل المعيقة لفعالية مراقبة الأحمال التدريبية وإدارتها؟

|  | لا يحد  من الفعاليه  1 | 2 | إلى حد ما  يحد الفعاليه  3 | 4 | يحد بشدة  من الفعاليه  5 |
| --- | --- | --- | --- | --- | --- |
| نقص في الأدوات |  |  |  |  |  |
| قلة الوقت / الموظفين |  |  |  |  |  |
| وعي المدرب وتقبله |  |  |  |  |  |
| لا يوجد إجماع على تحليل البيانات |  |  |  |  |  |
| صدق / ثبات / حساسية الاختبارات الميدانية |  |  |  |  |  |
