# Supplementary material for: Arabic translation and cultural adaptation of a training load and player monitoring in high-level football questionnaire: A cognitive interview study
Source: PLoS One. 2024 Apr 17;19(4):e0302006. doi: 10.1371/journal.pone.0302006 (PMC11023223; doi:10.1371/journal.pone.0302006)
Supplement: Attachment — Submitted filename: PONE-D-23-42649_reviewer.pdf [file pone.0302006.s002.pdf]

PLOS ONE

Arabic Translation and Cultural Adaptation of a Training Load And Player Monitoring in High-Level Football Questionnaire: A Cognitive Interview Study

--Manuscript Draft--

**Manuscript Number:**	PONE-D-23-42649
**Article Type:**	Research Article
**Full Title:**	Arabic Translation and Cultural Adaptation of a Training Load And Player Monitoring in High-Level Football Questionnaire: A Cognitive Interview Study
**Short Title:**	Cross-Cultural Adaptation of Player Monitoring Questionnaire
**Corresponding Author:**	Abdulmalek K. Bursais<br>KFU: King Faisal University<br>Alahsa, SAUDI ARABIA
**Keywords:**	Keywords: load Monitoring; sports performance; fitness profile, soccer, physical preparation
**Abstract:**	Background: Understanding the current practice and the associated challenges in applying monitoring tools is essential to improving football performance in the Middle East. Thus, the purpose was to translate and culturally adapt a published questionnaire that assessed the practice and perception of High-Level football teams toward Training Load and Player Monitoring to be used in the Arabic context. Method: A total of 15 Arabic Speaking coaches (mean age 42.6±9.9; mean experience 10.9±5.7; 53.3% football coaches, and 46.7% strength & conditioning coaches) have participated in this study. The current study followed a systematic cross-cultural adaptation process that involved forward translation, synthesis, back-translation, expert panel, and pre-testing (cognitive interviewing). Three rounds of cognitive interviews were held with 15 participants. Descriptive data were reported for characteristics. Result: With some minor modifications to the questionnaire by the expert committee, the instrument was translated and cross-culturally adapted into Arabic. 100% of the participants confirmed that the resulting Arabic versions of the training load and player monitoring in high-level football Questionnaire were appropriate. Conclusion: The training load and player monitoring in high-level football Questionnaire were successfully cross-culturally adapted into Arabic and are now ready for use in the Arabic context.
**Order of Authors:**	Abdulmalek K. Bursais
**Additional Information:**	
**Question**	**Response**
**Financial Disclosure**<br><br>Enter a financial disclosure statement that describes the sources of funding for the work included in this submission. Review the submission guidelines for detailed requirements. View published research articles from *PLOS ONE* for specific examples.<br><br>This statement is required for submission and **will appear in the published article** if the submission is accepted. Please make sure it is accurate.	Yes

Powered by Editorial Manager® and ProduXion Manager® from Aries Systems Corporation

**Funded studies**<br>Enter a statement with the following details:<br>• Initials of the authors who received each award<br>• Grant numbers awarded to each author<br>• The full name of each funder<br>• URL of each funder website<br>• Did the sponsors or funders play any role in the study design, data collection and analysis, decision to publish, or preparation of the manuscript?<br><br>Did you receive funding for this work?	
Please add funding details.<br>as follow-up to "**Financial Disclosure**<br><br>Enter a financial disclosure statement that describes the sources of funding for the work included in this submission. Review the submission guidelines for detailed requirements. View published research articles from *PLOS ONE* for specific examples.<br><br>This statement is required for submission and **will appear in the published article** if the submission is accepted. Please make sure it is accurate.<br><br>**Funded studies**<br>Enter a statement with the following details:<br>• Initials of the authors who received each award<br>• Grant numbers awarded to each author<br>• The full name of each funder<br>• URL of each funder website<br>• Did the sponsors or funders play any role in the study design, data collection and analysis, decision to publish, or preparation of the manuscript?<br><br>Did you receive funding for this work?"	This research was supported by the Deanship of Scientific Research, Vice Presidency for Graduate Studies and Scientific Research, King Faisal University, Saudi Arabia [Project No. GRANT1445].
Please select the country of your main research funder (please select carefully as in some cases this is used in fee calculation).	SAUDI ARABIA - SA

as follow-up to "**Financial Disclosure**<br><br>Enter a financial disclosure statement that describes the sources of funding for the work included in this submission. Review the submission guidelines for detailed requirements. View published research articles from *PLOS ONE* for specific examples.<br><br>This statement is required for submission and **will appear in the published article** if the submission is accepted. Please make sure it is accurate.<br><br>**Funded studies**<br>Enter a statement with the following details:<br>• Initials of the authors who received each award<br>• Grant numbers awarded to each author<br>• The full name of each funder<br>• URL of each funder website<br>• Did the sponsors or funders play any role in the study design, data collection and analysis, decision to publish, or preparation of the manuscript?<br><br>Did you receive funding for this work?"	
**Competing Interests**<br><br>Use the instructions below to enter a competing interest statement for this submission. On behalf of all authors, disclose any competing interests that could be perceived to bias this work—acknowledging all financial support and any other relevant financial or non-financial competing interests.<br><br>This statement is required for submission and **will appear in the published article** if the submission is accepted. Please make sure it is accurate and that any funding sources listed in your Funding Information later in the submission form are also declared in your Financial Disclosure statement.	The authors have declared that no competing interests exist.

Powered by Editorial Manager® and ProduXion Manager® from Aries Systems Corporation

View published research articles from *PLOS ONE* for specific examples.<br><br>**NO authors have competing interests**<br><br>Enter: *The authors have declared that no competing interests exist.*<br><br>**Authors with competing interests**<br><br>Enter competing interest details beginning with this statement:<br><br>*I have read the journal's policy and the authors of this manuscript have the following competing interests: [insert competing interests here]*<br><br>* typeset	
**Ethics Statement**<br><br>Enter an ethics statement for this submission. This statement is required if the study involved:<br><br>• Human participants<br>• Human specimens or tissue<br>• Vertebrate animals or cephalopods<br>• Vertebrate embryos or tissues<br>• Field research<br><br>Write "N/A" if the submission does not require an ethics statement.<br><br>General guidance is provided below. Consult the submission guidelines for detailed instructions. **Make sure that all information entered here is included in the Methods section of the manuscript.**	The study was conducted in accordance with the Declaration of Helsinki and approved by the In-stitutional Review Board of King Faisal University, Saudi Arabia (Reference number: KFU-REC-2023-MAR-ETHICS707, approved on 23 March 2023).

Powered by Editorial Manager® and ProduXion Manager® from Aries Systems Corporation

**Format for specific study types**<br><br>**Human Subject Research (involving human participants and/or tissue)**<br>• Give the name of the institutional review board or ethics committee that approved the study<br>• Include the approval number and/or a statement indicating approval of this research<br>• Indicate the form of consent obtained (written/oral) or the reason that consent was not obtained (e.g. the data were analyzed anonymously)<br><br>**Animal Research (involving vertebrate animals, embryos or tissues)**<br>• Provide the name of the Institutional Animal Care and Use Committee (IACUC) or other relevant ethics board that reviewed the study protocol, and indicate whether they approved this research or granted a formal waiver of ethical approval<br>• Include an approval number if one was obtained<br>• If the study involved *non-human primates*, add *additional details* about animal welfare and steps taken to ameliorate suffering<br>• If anesthesia, euthanasia, or any kind of animal sacrifice is part of the study, include briefly which substances and/or methods were applied<br><br>**Field Research**<br><br>Include the following details if this study involves the collection of plant, animal, or other materials from a natural setting:<br>• Field permit number<br>• Name of the institution or relevant body that granted permission	
**Data Availability**<br><br>Authors are required to make all data underlying the findings described fully available, without restriction, and from the time of publication. PLOS allows rare exceptions to address legal and ethical concerns. See the PLOS Data Policy and FAQ for detailed information.	Yes - all data are fully available without restriction

A Data Availability Statement describing where the data can be found is required at submission. Your answers to this question constitute the Data Availability Statement and **will be published in the article**, if accepted.<br><br>**Important:** Stating 'data available on request from the author' is not sufficient. If your data are only available upon request, select 'No' for the first question and explain your exceptional situation in the text box.<br><br>Do the authors confirm that all data underlying the findings described in their manuscript are fully available without restriction?	
**Describe where the data may be found in full sentences. If you are copying our sample text, replace any instances of XXX with the appropriate details.**<br><br>• If the data are **held or will be held in a public repository**, include URLs, accession numbers or DOIs. If this information will only be available after acceptance, indicate this by ticking the box below. For example: *All XXX files are available from the XXX database (accession number(s) XXX, XXX.).*<br>• If the data are all contained **within the manuscript and/or Supporting Information files**, enter the following: *All relevant data are within the manuscript and its Supporting Information files.*<br>• If neither of these applies but you are able to provide **details of access elsewhere**, with or without limitations, please do so. For example:<br><br>*Data cannot be shared publicly because of [XXX]. Data are available from the XXX Institutional Data Access / Ethics Committee (contact via XXX) for researchers who meet the criteria for access to confidential data.*<br><br>*The data underlying the results presented in the study are available from (include the name of the third party*	All relevant data are within the manuscript and its Supporting Information files.

and contact information or URL).<br>• This text is appropriate if the data are owned by a third party and authors do not have permission to share the data.<br><br>* typeset	
Additional data availability information:	

Click here to access/download;Manuscript;Manuscript.docx

Arabic Translation and Cultural Adaptation of a Training Load And Player Monitoring in High-Level Football Questionnaire: A Cognitive Interview Study

Abdulmalek K. Bursais[1,*]

[1] Department of Physical Education, College of Education, King Faisal University, Al-Ahsa 31982, Saudi Ara-bia; abursais@kfu.edu.sa

*Correspondence: abursais@kfu.edu.sa;

Abstract

Background

Understanding the current practice and the associated challenges in applying monitoring tools is essential to improving football performance in the Middle East. Thus, the purpose was to translate and culturally adapt a published questionnaire that assessed the practice and perception of High-Level football teams toward Training Load and Player Monitoring to be used in the Arabic context.

Method

A total of 15 Arabic Speaking coaches (mean age 42.6±9.9; mean experience 10.9±5.7; 53.3% football coaches, and 46.7% strength & conditioning coaches) have participated in this study. The current study followed a systematic cross-cultural adaptation process that involved forward translation, synthesis, back-translation, expert panel, and pre-testing (cognitive interviewing). Three rounds of cognitive interviews were held with 15 participants. Descriptive data were reported for characteristics.

Result

With some minor modifications to the questionnaire by the expert committee, the instrument was translated and cross-culturally adapted into Arabic. 100% of the participants confirmed that the resulting Arabic versions of the training load and player monitoring in high-level football questionnaires were appropriate.

Conclusion

The training load and player monitoring in high-level football questionnaires were successfully cross-culturally adapted into Arabic and are now ready for use in the Arabic context.

Keywords: Load Monitoring, Sports performance, Fitness profile, Soccer, Physical preparation

Introduction

In a bid to reduce the occurrence of injuries and enhance performance, numerous elite football teams enlist the services of fitness and sport-science experts who diligently oversee the daily monitoring of training load (TL) (1). TL is generally categorized into two components: external and internal training load. External training load refers to the physical exertion undertaken by the athlete, such as distance covered or number of sprints completed (2). Internal training load, on the other hand, pertains to the physiological response induced by the training, including factors like heart rate and the athlete's perceived effort (2). While there is evidence supporting the connection between training load and both performance and injury risk (3,4), there remains a limited understanding of how these monitoring methods are practically applied within the realm of football.

Advancements in technology and analytical techniques have ushered in new opportunities within the applied sports environment. Nowadays, sports practitioners have the capability to monitor training load (TL) through the utilization of global navigation satellite systems (GNSS) and other microtechnology (5,6). This contemporary technology yields a wide array of variables, allowing practitioners to dissect and quantify TL in more intricate detail than ever before. Nevertheless, there is currently no consensus regarding which variables are the most valuable or, indeed, how to effectively analyze the longitudinal data collected from a diverse roster of players (7). Similarly, there exists limited knowledge regarding the current practices and the associated challenges in applying these methods within professional football, primarily due to their insufficient representation in the published literature.

In the past, elite and professional teams might have been hesitant to share their methods, but recent research indicates their increasing willingness to participate in and publish applied research (8,9). Offering an overview of the prevailing practices and viewpoints on monitoring will shed light on the difficulties experienced by practitioners and will inspire more relevant research within the industry (10,11). The study by Akenhead & Nassis (7) offers probably the most comprehensive empirical analysis of information on the practices and practitioners' perceptions of monitoring in high-level professional football clubs. Their research revealed that factors such as human resources, low coach buy-in, and poor sensitivity of field measures have limited the effectiveness of training monitoring (7). While most of the research regards the training load was conducted in Europe, the United States, and Australasia, yet nothing has been known about the practice of football teams in the Middle East (especially in

Saudi Arabia). Therefore, the purpose of the current study is to translate and culturally adapt the Training Load and Player Monitoring in High-Level Football Questionnaire to be used in the Arabic context. This will help to de evelopment of representative samples and provide a set of standardized questionnaires for future use to compare data from different nations.

Methods

The present study was designed as a methodological cross-sectional investigation. To assemble a participant group, a convenience sampling method was utilized, resulting in a total of 15 Arab coach participants, all of whom were aged over 32 years old from different Arab countries. Recruitment efforts spanned diverse locations within the Arab Region and were facilitated through the distribution of an online flyer, disseminated via platforms such as X (previously Twitter) and WhatsApp. Additionally, coaches working in Saudi Arabia during the period from July to September 2023 were approached for participation. Notably, individuals lacking fluency in the Arabic language were excluded from the study. This study was approved by the Institutional Review Board at King Faisal University, Alahsa, Saudi Arabia (IRP: ETHICS707), and participants provided written consent for their involvement.

Instrument

This research details the comprehensive procedure involved in translating and culturally modifying the survey into Arabic for use with Arabic-speaking coaches. The original survey contained a total of 15 questions, encompassing 9 open-ended questions and 6 closed-ended

ones. These questions pertained to various aspects such as the aims and methodologies of monitoring, analysis of data, and evaluations of the effectiveness of player monitoring. The instrument was made accessible on the International Journal of Sports Physiology and Performance's platform (7).

Cross-cultural adaptation process

The present study adhered to the guidelines outlined for cross-cultural adaptation studies as recommended by (12), and was adapted in a previous study (13). The process is illustrated in Figure 1.

Figure 1 Illustrates the translation and cross-cultural adaptation process as recommended by Beaton, et al 2000.

1- Forward translation.

Two proficient and unbiased bilingual translators proficient in both English and Arabic were engaged to conduct the initial translation of the English versions of the survey. Translator 1 possessed a familiarity with the subjects and themes addressed within the questionnaire. In contrast, Translator 2 was deliberately kept uninformed about the specific concepts being measured. This adherence to the guidelines ensured that Translator 2 ideally had no prior knowledge in the related field (12).

2- Synthesis of the translation.

The author thoroughly reviewed the translated questionnaires generated in the initial phase, addressing and rectifying any discrepancies encountered. Following this, the author, along with the two translators, collaborated to generate a conclusive and mutually accepted version for each questionnaire, resulting in the creation of the final agreed-upon document known as the T12 questionnaire.

3- Back translation.

The process of back translation for the T12 questionnaires was performed by two distinct bilingual independent translators. In this procedure, the translators retranslated the T12 questionnaire from the target language back into the original language (from Arabic to English), all while remaining unaware of the initial questionnaire or its subject matter. This back-translation phase serves the purpose of confirming that the questions are lucid, comprehensible, and accurately translated. The primary motivation behind this step was to prevent any potential information bias and uncover unforeseen nuances in the phrasing of items within the translated questionnaire, i.e., the T12 version.

4- Expert committee.

The panel of experts consisted of five specialists well-versed in the domain of the instruments' knowledge and translation processes (forward and back translators). All members of the expert committee held doctoral degrees, possessed bilingual proficiency (Arabic-English),

and boasted substantial expertise in player monitoring, strength & conditioning, coaching, exercise physiology, sports science, and linguistics. The composition of this expert committee played a pivotal role in ensuring the achievement of satisfactory cross-cultural alignment within the research tools.

The complete set of original questionnaires, along with each translation (forward and backward), alongside corresponding written assessments composed by the author, were distributed to each expert committee member for thorough review. Their input was crucial in making informed judgments and validating the cross-cultural consistency between the original version and the final versions according to the guideline (12).

5- Pre-testing version.

Fifteen coaches specializing in soccer or strength & conditioning, all of whom were native Arabic speakers (ages ranging from 30 to 65 years), participated in the completion of the questionnaires. Additionally, they engaged in cognitive interviews aimed at ascertaining their interpretations of the items within each questionnaire and the corresponding responses they selected, as per the provided definitions. The objective behind this pretesting phase of the novel questionnaire was to employ the pre-final version on participants within the intended context.

Cognitive interviews

A cognitive interview serves as a qualitative technique designed specifically to examine the extent to which a survey question effectively accomplishes its intended purpose (14). These cognitive interviews were conducted through face-to-face interactions or Zoom meetings, each held separately subsequent to a participant's completion of the final Arabic rendition of the translated questionnaires. Three rounds of cognitive interviews were conducted; five participants were involved in each round. These cognitive interviews aimed to validate that the respondents comprehended the questionnaire items and identify any potential need for rephrasing or restructuring. All interview queries were semi-structured and open-ended, conducted entirely in Arabic. The role of the interviewer was assumed by the author in this context. The duration taken to address the questions was recorded. In each round, adjustments were made to the questions based on the feedback received from the participants. The subsequent content outlines the specific questions posed to participants during these cognitive interviews:

1. What do you think the question is about?
2. Is the question clear and understandable? If not, how can it be made clearer?
3. Do you have any questions about the items?
4. How could the wording be clearer?

Additional overall questions:

1. Are there any activities or examples that we omitted?
2. Did any of the questions make you feel uncomfortable?

Statistical analysis

Descriptive data analysis was conducted for the study. Continuous variables were summarized using means along with their corresponding standard deviations (SD). For the cognitive interviews, the overall total score for each round was calculated as follows: The number of responses (each question in the cognitive interview) multiplied by 100, divided by the total number of answers. This provided the percentage of responses that were not flagged as having issues with their meaning. All data analyses were executed using IBM's SPSS version 27 (version 27.0; SPSS, Inc., Chicago, Illinois).

Table 1 Summary characteristics of the sample population (n = 15).

**Variables**	**R1 (n=5)**	**R2 (n=5)**	**R3 (n=5)**
	**Mean + SD**		
**Age (Years)**	37 ± 4.8	39.2 ± 9.4	51.6 ± 8.9
**Experience (Year)**	6 ± 8	10.6 ± 4.8	15.2 ± 6
**Coaching Type**			
**Football Coach**	2	3	3
**Strength & Conditioning Coach**	3	2	2
**Education**			
**High School**	2		
**College Degree**	2	2	2

**Post-gradute Degree**	1	3	3
**Empolyment Status**			
**Unempolyed**	1		1
**Part-time Coach**	2	2	3
**Full-time Coach**	2	3	1
**Nationality**			
**Saudi**	2	3	3
**Egyptian**	2	1	1
**Tunisian**	1	1	1

R = round, SD = standard deviation

Results

Cross-cultural adaptation

Forward translation and synthesis of the translation. Starting from the original survey, two separate skilled bilingual translators effectively converted the questionnaires into Arabic. Some slight differences arose due to the direct translation of certain English terms into Arabic. These disparities were addressed by the author and translators in the initial translation stage, leading to the creation of the T12 questionnaire version.

Backward translation. The T12 version of the questionnaire was translated from Arabic to English by two distinct independent bilingual translators. This process served as a means to

ensure its validity; however, slight adjustments were identified within the questionnaire and subsequently resolved.

Expert committee. The Five experts reviewed the original questionnaires and the translated questionnaires together with the corresponding written reports provided by the author. The expert committee provided advice on necessary cultural modifications to the questionnaire items. For example, the Arabic language is not dominant in the leagues mentioned in the original version of the survey. Consequently, the experts proposed alternative leagues with Arabic dominance. Also, the expert panel suggested a few minor changes that could better align with the Arabic culture where, as an example, "*Sport Scientist*" was changed to "*Sport Science Specialist*". Table 2 details the minor changes provided by the expert committee, this alteration is acknowledged within the literature (13).

Table 2 Minor changes provided by the expert committee of the training load and player monitoring in high-level football questionnaires Arabic version

	**Original item in the English version**	**Modified item in the Arabic version**
**1**	*Sport Scientist*	*Sport Science Specialist*
**2**	*Strength & Conditioning Coach*	*Muscular Strength and Physical Preparation Coach*
**3**	*Rate of Perceived Exertion (RPE)*	*Borg's Perceived Exertion Scale*

Cognitive interview. Fifteen football and strength & conditioning coaches who were native Arabic speakers aged between 32 and 55 years old participated in the interviews. The interviews

consisted of three rounds of cognitive assessments, with five participants in each round responding to the questionnaires. The findings from these cognitive interviews conducted in Arabic are detailed in Table 3 (provided below). It was found that across all three rounds, all participants comprehended the intended meanings of the questionnaire items, achieving a 100% understanding rate. The collective outcomes underscore that every participant (100%) grasped the intended interpretations of the Arabic version's items throughout all rounds.

Table 3 Overall result of cognitive interview of the training load and player monitoring in high-level football questionnaires Arabic version

Rounds	N	Participant Understanding of the Intended Meaning	The Content Was Clear for the Participant	The Wording Was Clear for the Participant
**R1**	5	100%	100%	100%
**R2**	5	100%	100%	100%
**R3**	5	100%	100%	100%

Discussion

The present study has effectively accomplished the translation of the instrument from English into Arabic. The research instruments underwent a process of translation and cultural adaptation to guarantee equivalence between the original versions and the versions created in

this study, as stipulated by guideline (12). The availability of a valid Arabic-language version of the questionnaire for use with Arabic-speaking coaches holds significant importance for research conducted in Middle Eastern countries, given the substantial prevalence of football interest in these regions. In summary, the outcomes affirm that the new version has been successfully translated into Arabic and culturally adjusted to align with the Arabic context.

Although this study has successfully demonstrated that the translated Questionnaire is a valid and reliable instrument for use with Arabic-speaking coaches, this study has several limitations. First, while we tried to reach as many Arabic-speaking coaches to assess the reliability, only 15 coaches participated. Second, while there are 22 Arabic countries, only individuals from three countries (Saudi Arabia, Egypt, and Tunisia) participated. This may limit the understanding of the questionnaires for other Arabic countries due to some cultural differences. This instrument therefore needs to be interpreted with caution when applied in different Arab countries. Future research may investigate whether this instrument would be valid to be used with Arabic-speaking coaches from all over the Arabic region.

Acknowledgments

The author would like to express his gratitude to all participants, translators, and the expert committee for their contributions to the cross-cultural process. The author would like to thank the Deanship of Scientific Research, Vice Presidency for Graduate Studies and Scientific

Research, King Faisal University, Saudi Arabia for the financial support under Ambitious Researcher track [Project No. GRANT1445].

Funding

This research was supported by the Deanship of Scientific Research, Vice Presidency for Graduate Studies and Scientific Research, King Faisal University, Saudi Arabia [Project No. GRANT1445].

References

1. McCall A, Davison M, Andersen TE, Beasley I, Bizzini M, Dupont G, et al. Injury prevention strategies at the FIFA 2014 World Cup: perceptions and practices of the physicians from the 32 participating national teams. Br J Sports Med. 2015 May;49(9):603–8.

2. Wallace LK, Slattery KM, Coutts AJ. A comparison of methods for quantifying training load: relationships between modelled and actual training responses. Eur J Appl Physiol. 2014 Jan;114(1):11–20.

3. Eirale C, Tol JL, Farooq A, Smiley F, Chalabi H. Low injury rate strongly correlates with team success in Qatari professional football. Br J Sports Med. 2013 Aug 1;47(12):807–8.

4. Hägglund M, Waldén M, Magnusson H, Kristenson K, Bengtsson H, Ekstrand J. Injuries affect team performance negatively in professional football: an 11-year follow-up of the UEFA Champions League injury study. Br J Sports Med. 2013 Aug 1;47(12):738–42.

5. Bursais AK, Bazyler CD, Dotterweich AR, Sayers AL, Alibrahim MS, Alnuaim AA, et al. The Relationship between Accelerometry, Global Navigation Satellite System, and Known Distance: A Correlational Design Study. Sensors. 2022 Apr 27;22(9):3360.

6. Principe VA, Vale RG de S, Nunes R de AM. A systematic review of load control in football using a Global Navigation Satellite System (GNSS). Mot Rev Educ Física. 2020;26(4):e10200059.

7. Akenhead R, Nassis GP. Training Load and Player Monitoring in High-Level Football: Current Practice and Perceptions. Int J Sports Physiol Perform. 2016 Jul;11(5):587–93.

8. Malone JJ, Michele RD, Morgans R, Burgess D, Morton JP, Drust B. Seasonal Training-Load Quantification in Elite English Premier League Soccer Players. Int J Sports Physiol Perform. 2015 May 1;10(4):489–97.

9. Thorpe RT, Strudwick AJ, Buchheit M, Atkinson G, Drust B, Gregson W. Tracking Morning Fatigue Status Across In-Season Training Weeks in Elite Soccer Players. Int J Sports Physiol Perform. 2016 Oct 1;11(7):947–52.

10. Bourdon PC, Cardinale M, Murray A, Gastin P, Kellmann M, Varley MC, et al. Monitoring Athlete Training Loads: Consensus Statement. Int J Sports Physiol Perform. 2017 Apr;12(s2):S2-161-S2-170.

11. Cardinale M, Varley MC. Wearable Training-Monitoring Technology: Applications, Challenges, and Opportunities. Int J Sports Physiol Perform. 2017 Apr;12(s2):S2-55-S2-62.

12. Beaton DE, Bombardier C, Guillemin F, Ferraz MB. Guidelines for the Process of Cross-Cultural Adaptation of Self-Report Measures: Spine. 2000 Dec;25(24):3186–91.

13. Alaqil AI, Gupta N, Alothman SA, Al-Hazzaa HM, Stamatakis E, Del Pozo Cruz B. Arabic translation and cultural adaptation of sedentary behavior, dietary habits, and preclinical mobility limitation questionnaires: A cognitive interview study. Farghaly SM, editor. PLOS ONE. 2023 Jun 12;18(6):e0286375.

14. Willis G. Analysis of the Cognitive Interview in Questionnaire Design [Internet]. Oxford University Press; 2015 [cited 2023 Sep 6]. Available from: https://global.oup.com/academic/product/analysis-of-the-cognitive-interview-in-questionnaire-design-9780199957750?cc=sa&lang=en&

Figure 1

Click here to access/download;Figure;Figure 1 Illustrates the translation and cross.docx

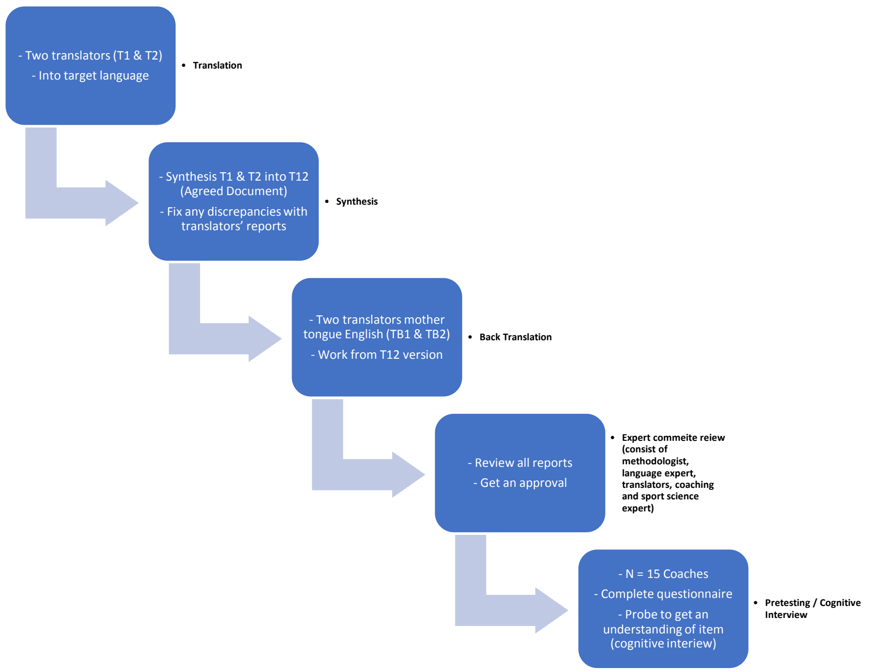

Figure 1 Illustrates the translation and cross-cultural adaptation process as recommended by Beaton, et al 2000.

The translated questionnaire

Click here to access/download
Supporting Information
Translated Questionnaire.docx
