# Supplementary material for: Arabic translation and cultural adaptation of a training load and player monitoring in high-level football questionnaire: A cognitive interview study
Source: PLoS One. 2024 Apr 17;19(4):e0302006. doi: 10.1371/journal.pone.0302006 (PMC11023223; doi:10.1371/journal.pone.0302006)
Supplement: Attachment — Submitted filename: Response to Reviewers.docx [file pone.0302006.s005.docx]

26 February 2024

**Dear Dr. Elsahoryi,**

I would like to submit our revised manuscript (submission ID: PONE-D-23-42649) entitled “Arabic Translation and Cultural Adaptation of a Training Load And Player Monitoring in High-Level Football Questionnaire: A Cognitive Interview Study” for review and consideration for publication in PLOS ONE.

I would like to thank the editor and the reviewers for providing constructive feedback on my manuscript. I have addressed each comment individually below. I uploaded a copy of the manuscript with the changes tracked in addition to the manuscript file with all changes accepted. I believe these changes have strengthened the paper, and I look forward to the next steps in the review process.

The funders had no role in study design, data collection and analysis, decision to publish, or preparation of the manuscript."

**Sincerely,**

**Abdulmalek Bursais**

**Reviewer Comments:**

| **Reviewer 1 comments** | **Response to reviewer 1** |
| --- | --- |
| What is the rationale of the study? What are the objectives and gap to address? Why is this the purpose of the study? | Thank you for your comment, this has been addressed in the revised manuscript. |
| The paper can make use of professional copyediting. | Thank you for your feedback. I acknowledge the suggestion for professional copyediting and will ensure that the manuscript undergoes thorough review and editing to enhance its clarity, coherence, and overall readability. |
| How were the participants selected? | This has been edited in the revised manuscript as participants were conveniently selected. |
| This is not clear. Please explain. | Done |
| What were the modifications and why? | Thank you for your feedback. this has been added to the revised manuscript. |
| What were the translation challenges and the cultural aspects met during the translation? Who did the translation? On what basis did the participants confirm that the translation was appropriate? | Thank you for your feedback. this has been clarified in the revised manuscript. |
| This is a repetition of the result. What is the actual conclusion in relation to the study rationale and objectives. What are the implications/recommendations. | Thank you for your feedback. this has been fixed in the revised manuscript. |
| Soccer or football? Check consistency. | Has been changed to football |
| Translation | Fixed |
| If this is the gap the study attempts to address, how come the purpose of the study is to translate a questionnaire? It is never mentioned that language is the problem that led to having limited knowledge or having no consensus and therefore there is an attempt to translate the questionnaire to help solve this problem. The problem mentioned here is lack of data regarding the variables and data analysis of player performance and the challenges of applying these practices. | Thank you for your feedback. The introduction has been fixed to better describe the gap the study attempts to address and to better describe the objective of the current study. |
| Only one study for the literature review? More studies are needed, especially regarding the core of this study which is the translation and cultural adaptation of foreign training material into Arabic. | Added |
| Cite these studies. | Done |
| This means that the study will investigate the performance of football teams in Saudi Arabia, which is not the case here since translating a questionnaire does not tell much about the practice of football in Saudi Arabia or anywhere. | Thank you for your feedback. this has been fixed in the revised manuscript. |
| This purpose does not seem to align with all the build-up of the Introduction. It is unclear why we are translating the questionnaire and how. According to the title, the translation process is the core of this research, but this does not show in the Introduction which never mentions translation or language except here. | Thank you for your feedback. This has been fixed to better describe the purpose of the current study and better align with all the build-up of the Introduction in the revised manuscript. |
| Justify. | Done |
| This is not open-ended. It is a close-ended yes-no question. | Fixed |
| This is not open-ended. It is a close-ended yes-no question. | Fixed |
| This is not open-ended. It is a close-ended yes-no question. | Fixed |
| This is not open-ended. It is a close-ended yes-no question. | Fixed |
| Elaborate. | Done |
| How? | Added |
| How? | Added |
| Elaborate | Done |
| Elaborate | Done |
| This column should have both the Arabic and its translation. | Thank you for your suggestion, this has been added. |
| Your interpretation/discussion of the results should be elaborated and connected to both related previous studies and your research questions/objectives. What is written here is more of a conclusion and not a discussion. | Thank you for your feedback. The discussion has been fixed to better discuss the results and objectives of the study and connected to the related previous studies. |
| How? Standard Arabic is understood by all Arab countries alike. This point is not logical. | Thank you for your feedback. this has been fixed in the revised manuscript. |
| Same as previous comment. | Thank you for your feedback. this has been fixed in the revised manuscript. |
| **Reviewer 2 comments** | **Response to reviewer 2** |
| The manuscript is considered technically acceptable to the journal and supports the data and conclusions stated.  The manuscript is considered scientifically and technically sound. The experimentation process is sound and a modern idea.  Perform statistical analysis fairly appropriately.  The data provided is correct.  According to my humble experience, the manuscript is presented in a clear manner and written in, I believe, sound language. | I thank the reviewer for the encouragement and thoughtful comments. |
